# ‘Peer pressure’ in larval *Drosophila*?

**DOI:** 10.1242/bio.20148458

**Published:** 2014-06-06

**Authors:** Thomas Niewalda, Ines Jeske, Birgit Michels, Bertram Gerber

**Affiliations:** 1 Leibniz Institut für Neurobiologie (LIN), Abteilung Genetik von Lernen und Gedächtnis, Brenneckestrasse 6, 39118 Magdeburg, Germany; 2 Universität Leipzig, Institut für Biologie, Genetik, Talstrasse 33, 04103 Leipzig, Germany; 3 Otto von Guericke Universität Magdeburg, Institut für Biologie, Verhaltensgenetik, Universitätsplatz 2, 39106 Magdeburg, Germany; 4 Center of Behavioural Brain Science (CBBS), Universitätsplatz 2, 39106 Magdeburg, Germany

**Keywords:** Olfaction, Learning, Memory, Behaviour, Social interaction

## Abstract

Understanding social behaviour requires a study case that is simple enough to be tractable, yet complex enough to remain interesting. Do larval *Drosophila* meet these requirements? In a broad sense, this question can refer to effects of the mere *presence* of other larvae on the behaviour of a target individual. Here we focused in a more strict sense on ‘peer pressure’, that is on the question of whether the behaviour of a target individual larva is affected by *what* a surrounding group of larvae is doing. We found that innate olfactory preference of a target individual was neither affected (i) by the level of innate olfactory preference in the surrounding group nor (ii) by the expression of learned olfactory preference in the group. Likewise, learned olfactory preference of a target individual was neither affected (iii) by the level of innate olfactory preference of the surrounding group nor (iv) by the learned olfactory preference the group was expressing. We conclude that larval *Drosophila* thus do not take note of specifically *what* surrounding larvae are doing. This implies that in a strict sense, and to the extent tested, there is no social interaction between larvae. These results validate widely used en mass approaches to the behaviour of larval *Drosophila*.

## INTRODUCTION

An appreciation of what the conspecifics are doing is fundamentally important for the organization of behaviour. This is so from the subtleties of peer pressure in humans, via coordinated hunting in wolves, penguins, or sharks, the intricate interactions in social bees, wasps and termites or the swarming of locusts, to the rituals of courtship in their respective form throughout the animal kingdom. Understanding these processes, however, faces a dilemma: a study case is needed that is both simple enough to be experimentally tractable, and complex enough to remain interesting. We wondered whether one compromise case can be larval *Drosophila*, because these animals have a brain with only about 10.000 neurons, which can be manipulated one at a time. Despite this numerical simplicity of their brains, the behaviour of *Drosophila* larvae is not hard-wired (Gerber and Stocker, 2007; Diegelmann et al., 2013; Schleyer et al., 2013): for example, presenting an odour with a food reward establishes associative memory that is specific for the kind and the intensity of the trained odour. However, it is unclear to which degree in this type of assay a given individual target larva is affected by what a surrounding group of larvae is doing. We ventured into an analysis of this question regarding olfactory behaviour. Two features of the employed tasks should be emphasized:

First, we ask for the significance of specifically *what* a group of larvae is doing to an embedded target individual. This is a question distinct from the equally interesting and important one asking whether the mere *presence* (or past-presence) of conspecifics affects an individual's behaviour (see Discussion).

Second, our experiments distinguish between innate and learned olfactory behaviour. This is because innate and learned olfactory behaviour employ distinct second-order ascending pathways either allowing an integration with reward signalling or not, and also differing in the level of integration along descending pathways. That is, in insects the olfactory sensory neurons target the antennal lobe where they synapse onto largely local interneurons as well as first-order projection neurons. These projection neurons, significantly, bifurcate and thus have two ascending target areas: one branch of the projection neurons targets the lateral protocerebrum and downstream premotor circuitry. This pathway is largely sufficient for innate olfactory behaviour (Heimbeck et al., 2001). The second branch takes a detour via the mushroom body, allowing integration with ascending reinforcement signals; it is arguably via this mushroom body loop that learned olfactory behaviour is organized (Michels et al., 2011). For the current context, it is important that these two pathways also differ in integrative function along the descending pathways. After odour-reward learning, manipulating the value of the test situation such that it is less, equal, or higher than the value of the training-reward revealed that learned olfactory behaviour depends on a comparison of both these values (Gerber and Hendel, 2006; Schleyer et al., 2011): learned olfactory behaviour is expressed only if the value of the memorized reward is higher than the value of the testing situation – that is, a larva tracks down the learned odour only if it expects a gain from doing so. Such organization of learned olfactory behaviour thus features an integrative descending processing stage at which the testing situation is considered by the larvae to decide whether to express learned behaviour – or not. In contrast, innate olfactory behaviour lacks this integrative organization and rather is executed in a reflex-like way. Therefore, when asking whether social cues are integrated into the organization of olfactory behaviour it is warranted to separately consider learned and innate behaviour.

## MATERIALS AND METHODS

### General

#### Flies

The *Drosophila melanogaster* wild-type strain Canton-Special (WT) and the *Orco^1^* mutant (a loss-of-function allele of the *Orco* gene [CG10609]) (Larsson et al., 2004; Vosshall and Hansson, 2011) were maintained on standard food in mass culture at 25°C, 60–70% relative humidity and a 13/11 hour light/dark cycle. The experimental generation of the cultures was separated into two types of vials, one with standard food and one dyed by adding five drops (approximately 0.25 ml) of red food colour (Erdbeerrot L1435, Ruth GmbH & Co. KG, Bochum, Germany) to approximately 30 ml of food medium. This procedure allowed us to discern pale, not dyed larvae from dyed larvae by the colouration of their gut.

Third instar larvae in the feeding stage aged five days after egg-laying were used. Larvae were collected from the food medium immediately before the experiment and gently rinsed in tap water for cleaning. Experiments were performed at room temperature under standard laboratory light, on a clean bench or under a fume hood. All experiments comply with applicable law.

#### Petri dishes

For preparation of PURE Petri dishes a thin layer of freshly boiled 1% agarose solution (electrophoresis grade, Carl Roth GmbH, Karlsruhe, Germany; using deionized water) was poured into a Petri dish of 85 mm inner diameter (Sarstedt, Nümbrecht, Germany). After solidifying, the Petri dishes were covered and stored at 4°C for use within the following week. For preparation of FRU Petri dishes, the procedure was the same, except that fructose (CAS: 57-48-7; purity 99%, Carl Roth GmbH, Karlsruhe, Germany; 36 g/100 ml [2 mol]) was added after boiling.

#### Odours

The odours *n*-amylacetate (AM; CAS: 628-63-7; Sigma-Aldrich, St Louis, USA; applied diluted 1:50 in paraffin oil CAS: 8012-95-1; Merck, Darmstadt, Germany) and 1-octanol (OCT; CAS: 111-87-5; Merck, Darmstadt, Germany) were used. To present odours to the larvae without gustatory contact, 10 µl of either odour was filled in custom-made teflon containers with an inner diameter of 5 mm. The containers were then covered with a perforated lid (seven 0.5 mm holes) for effective evaporation.

### Experimental principle

In principle we compare olfactory preference between equally-treated individual ‘target’ larvae of the same genotype that are embedded in differently behaving groups. In this way we test whether individuals are affected by *what* the group of larvae around it is doing. As it was unclear whether a thus defined ‘peer-pressure’ effect, if any, would manifest as increase or decrease in olfactory preference, we intended to work at moderate levels of preference throughout.

Given that a number of neuronal and behavioural differences between innate and learned olfactory behaviour exist (see Introduction), we separately consider innate olfactory behaviour, in the sense of ‘experimentally naïve’, and learned olfactory behaviour (Table 1).

**Table 1.**
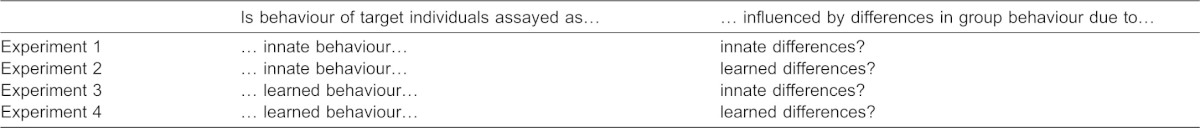
Overview of questions asked by Experiments 1–4

### Experiment 1

**Fig. 1.**
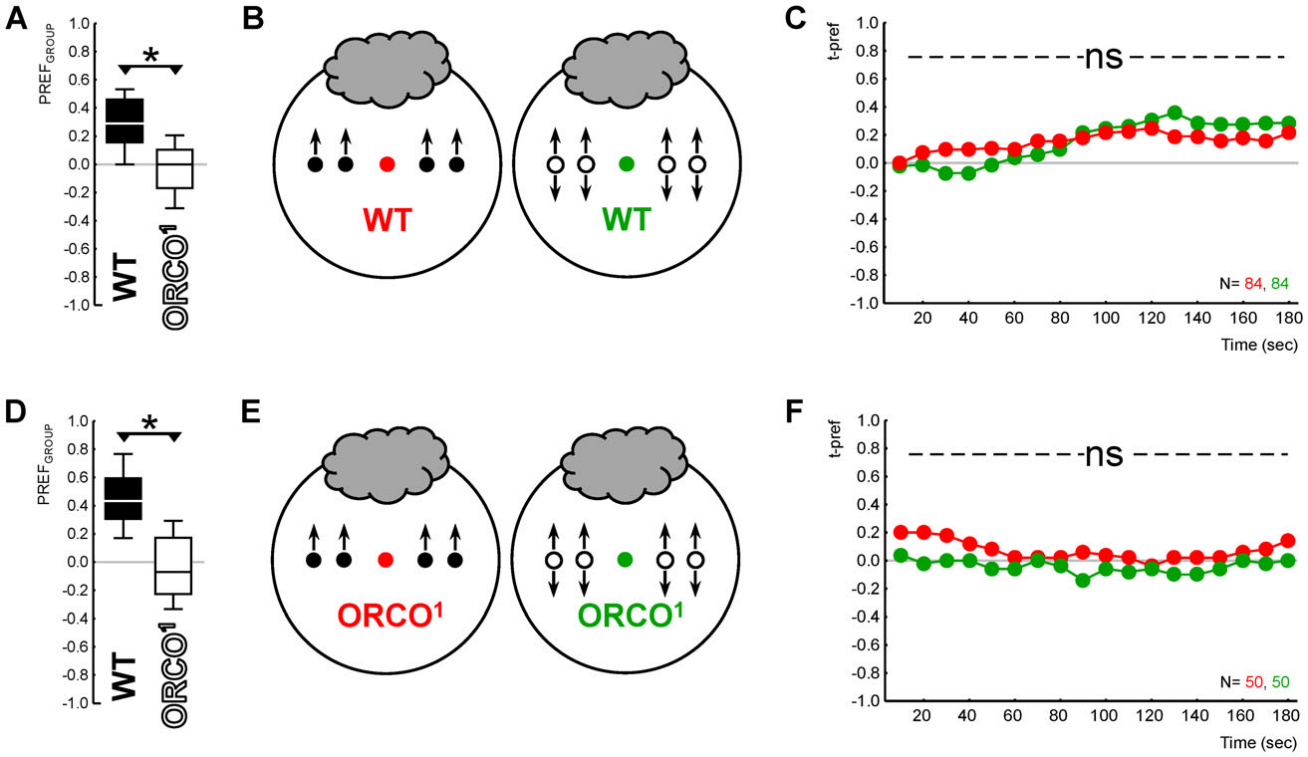
Innate olfactory behaviour of target individuals unaffected by innate behaviour of peer group. (A) Genetic differences in group behaviour: groups of WT larvae showed attraction for the odour (filled plot), while *Orco^1^* mutant groups did not (open plot); please note that the PREF_GROUP_ scores were taken three minutes after assay onset. Box plots show the median as the middle line, the 25%/75% quartiles as the box boundaries, and 10%/90% quantiles as the whiskers. (B) Sketch of the experimental principle: WT target individuals (coloured symbols) were embedded into either a WT group (filled symbols) or an *Orco^1^* group (open symbols). (C) Olfactory preference of the WT target individuals did not differ when embedded into either a smell-competent WT group (as is the case for the red symbols) or into a smell-blind, randomly behaving *Orco^1^* group (green symbols). Please note t-pref scores were taken every ten seconds, for a total of three minutes. (D–F) Likewise, *Orco^1^* target individuals behaved indistinguishably when embedded in smell-competent WT and in smell-blind *Orco^1^* groups. Note that panels A and D, respectively, show the behaviour of those groups of larvae sketched in panels B and E. All animals in this experiment were experimentally naïve; the grey cloud indicates the odour AM. The symbols * and ns, respectively, indicate P<0.05 or P>0.05 in MWU tests.

Olfactory preference was measured in single experimentally naïve larvae embedded either in a group of 29 WT larvae or in a group of 29 *Orco^1^* larvae that likewise were experimentally naïve. Since odours are typically attractive to WT larvae (e.g. Saumweber et al., 2011), we expected the WT group to prefer the odour, while the *Orco^1^* group, lacking an obligatory co-receptor for the function of *Or*-receptors (Larsson et al., 2004) and thus anosmic, should distribute randomly; this is indeed the case (Fig. 1A,D). Thus, the target individuals were embedded in groups that either showed olfactory preference (WT group), or not (*Orco^1^* group). Any difference in behaviour between the target individuals therefore would be due to the differential in *what* the WT versus the *Orco^1^* groups are doing.

We used either WT or *Orco^1^* larvae as target individuals, resulting in four individual–group combinations. This experimental design thus allowed us to ask whether a WT target individual is affected by the random behaviour of the anosmic *Orco^1^* mutant group around it (Fig. 1B), and in turn whether an anosmic *Orco^1^* mutant target individual uses non-olfactory cues to behave according to the WT group it is embedded in (Fig. 1E).

Measurements of olfactory preference followed standard methods. In brief, the target individual and the larvae comprising the group were collected from differentially dyed food vials. A PURE Petri dish was prepared by placing odour containers loaded with either diluted AM or diluent only (empty: EM) on opposite sides of the dish, creating a choice situation. Sidedness of odour placement was alternated across repetitions. Then, individual and group were placed onto the Petri dish, at about the midline between the odour containers; then the Petri dish was covered with a lid perforated in the middle with 15 one-mm holes to improve aeration.

To see whether as intended the olfactory behaviour of the WT group versus the *Orco^1^* mutant group differed, their AM preference was determined after the larvae had moved about the Petri dish for 3 min. From all our previous work, this is the time point where typically strongest odour preference can be observed: it is late enough to allow sufficient time to distribute, yet is early enough to not be influenced by e.g. adaptation. We counted the number of larvae (excluding the individual target larva, of course) located on the AM side (@_AM_), on the EM side (@_EM_) and along a 1 cm wide middle strip (@_MIDDLE_) and calculated preference as:
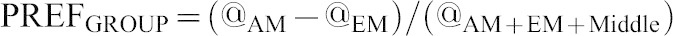
(1)

A positive PREF_GROUP_ value [−1; 1] thus indicates preference for AM, while avoidance of AM would show by negative values.

Given that in this as well as in all other experiments the olfactory behaviour of the respective groups did differ, it made sense to ask whether the behaviour of individual target larvae embedded in these groups would differ as well. Towards this end, the position of the target individuals was recorded every 10 seconds over the 3 min test period, resulting in eighteen observation time points. Following the convention introduced above for the group, a score of pref = 1 was assigned if the individual was located on the AM side, while when located on the EM side a score of pref = −1 was assigned; when located in the middle, a score of pref = 0 was recorded. To visualize the behaviour of the individuals over time, these pref-scores were, for each time point (t), averaged across the N-number of individuals (i) as t-pref:
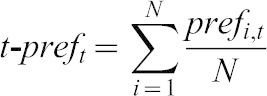
(2)

In addition we quantified, for each individual i, the degree of individual odour preference (i-pref) by summing up its pref scores across the eighteen observation time points for this respective individual and dividing by eighteen:
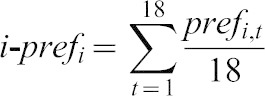
(3)

### Experiment 2

**Fig. 2.**
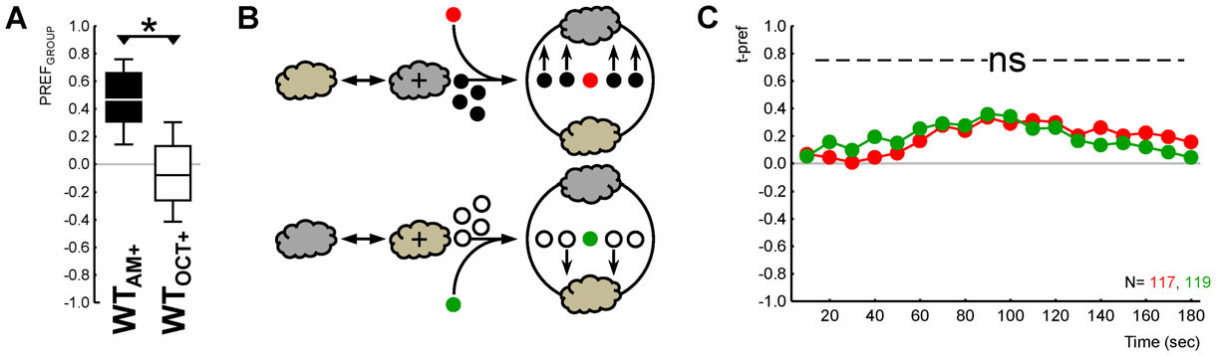
Innate olfactory behaviour of target individuals unaffected by learned behaviour of peer group. (A) Learned differences in group behaviour: groups of AM-rewarded larvae showed strong attraction to AM (filled plot), while OCT-rewarded groups showed slight attraction to OCT (open plot) (for sketch of training see panel B). (B) Sketch of the experimental principle: target individuals (coloured symbols) were embedded in groups that had either received the odour AM in conjunction with reward and the odour OCT without reward (AM+/OCT: filled symbols) or that had undergone reciprocal training (AM/OCT+: open symbols). (C) Olfactory preference of target individuals (red symbols) embedded into a group that based on its training history preferred AM did not differ from the behaviour of target individuals (green symbols) embedded in a group that expressed a memory-based preference for OCT. All animals in this experiment were of the smell-competent WT genotype. The grey cloud indicates the odour AM, the brown cloud the odour OCT. Other details are as in the legend for Fig. 1.

One WT group was rewarded upon presentation of AM but not of OCT, whereas the other WT group was rewarded reciprocally with OCT but not AM. Then, experimentally naïve WT target individuals were placed into these groups (Fig. 2B). Associative training, as intended, did lead to higher AM preference in the AM-rewarded as compared to the OCT-rewarded groups (Fig. 2A). Thus, any difference of olfactory behaviour between individual target larvae would indicate that it matters to them *what* the group around them was doing.

Associative training followed standard methods (Gerber et al., 2013). In brief, 30 WT larvae were collected from the food vial and placed onto a PURE Petri dish equipped with e.g. two OCT containers placed on opposite sides. Larvae were left crawling for 5 min. Then, animals were transferred onto a FRU Petri dish equipped with AM containers for 5 min. This training cycle (OCT/AM+) was repeated 3 times in total. For every trial fresh Petri dishes were used. Independent groups of 30 larvae were trained reciprocally (AM/OCT+) (note that the sequence of trial types was balanced over repetitions of the experiment [OCT/AM+ and AM+/OCT, or in the reciprocal case OCT+/AM and AM/OCT+]).

For testing, an experimentally naïve WT target individual from a differentially labelled food vial was added to the group. All larvae together were placed onto a PURE Petri dish with AM and OCT containers positioned on opposite sides to create a choice situation. Olfactory preference of the group and of the individuals was determined according to what was detailed above for Experiment 1 (please note that for Experiment 2 “EM” needs to be replaced by “OCT” in the respective expressions).

### Experiment 3

**Fig. 3.**
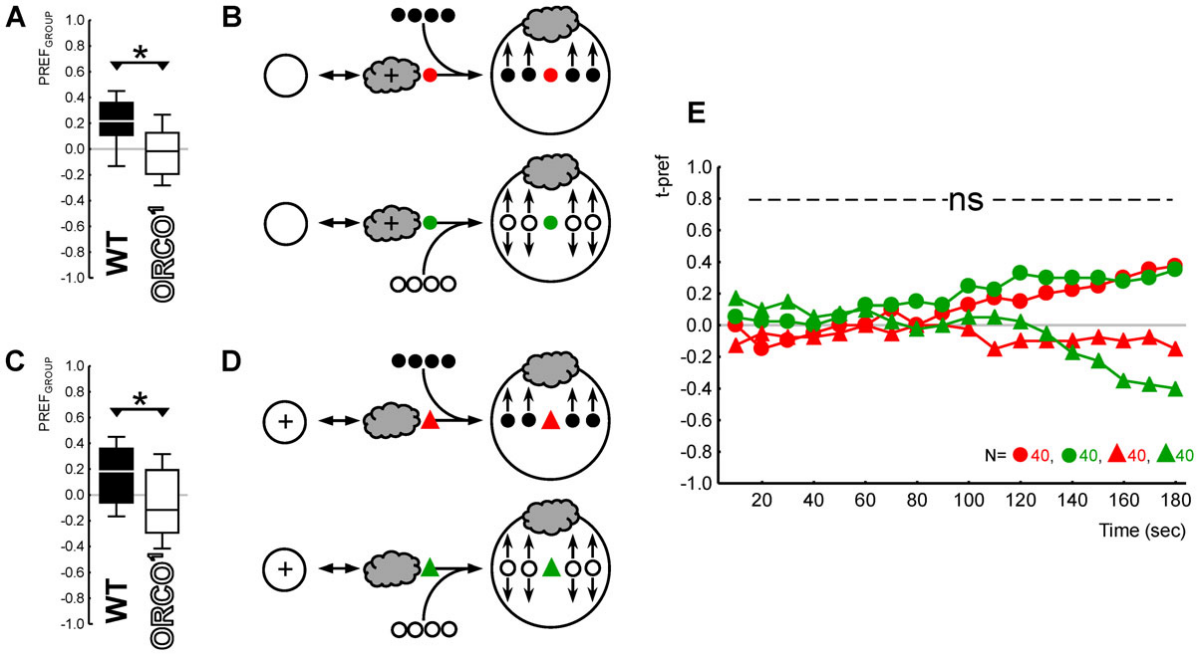
Learned olfactory behaviour of target individuals unaffected by innate behaviour of peer group. (A,C) Genetic differences in group behaviour: groups of WT larvae showed attraction for the odour (filled plots), while *Orco^1^* mutant groups did not (open plots). Note that panels A and C, respectively, show the behaviour of those groups of larvae sketched in panels B and D. (B,D) Sketches of the experimental principle. (B) Target individuals (coloured symbols) that had received AM-rewarded training were tested either embedded in a group of smell-competent WT larvae (filled symbols) (this was the case for the target individuals symbolized by red circles) or in a smell-blind *Orco^1^* mutant group (open symbols) (green-circle target individuals). (D) Target individuals that had received AM in a non-rewarded way were tested either in a WT group or in a smell-blind *Orco^1^* mutant group (red and green triangles indicating the respective target individuals). (E) Olfactory preference of target individuals that had received AM-rewarded training (cycles) was indistinguishable when embedded in a smell-competent WT group (red cycles) versus in a smell-blind *Orco^1^* mutant group (green cycles). The same holds true for target individuals that had received AM in a non-rewarded way (red versus green triangles). The ns symbol refers to P>0.05 in MWU tests in both these respective ‘red versus green’ comparisons. The grey cloud indicates the odour AM. Other details are as in the legend for Fig. 1.

We next tested whether the expression of learned olfactory behaviour of a target individual is affected by the behaviour of the group in which it is embedded during testing. The difference of group behaviour was ensured by using either WT groups or anosmic *Orco^1^* mutant groups. Consequentially, as in Experiment 1, the testing situation featured a choice between an odour side and a no-odour side. This is because in a two-odour testing situation *both* WT and *Orco^1^* mutant groups may distribute equally between sides (the *Orco^1^* mutants because they are anosmic, and the WT larvae because they are indifferent for the offered odours at the chosen dilutions). Thus, training followed the one-odour paradigm introduced by Saumweber et al.: larvae were trained as in Experiment 2, but instead of OCT an empty container was presented (Saumweber et al., 2011). In other words, larvae were trained either by paired presentations of AM and reward, alternated with blank trials during which an empty container was presented, or in the reciprocal case were trained by unpaired presentations of AM and reward. Thus, trained individual target WT larvae (randomly selected from a pool of 30 trained animals; the remaining subjects were discarded) were added either to a group of 29 experimentally naïve WT larvae, or to a group of 29 likewise experimentally naïve anosmic *Orco^1^* mutant larvae (Fig. 3B,D). Then, olfactory preference scores were determined as described for Experiment 1. Also in this experiment, the target individual and the group were differentially labelled.

As expected, the WT group shows olfactory preference while the *Orco^1^* group distributes randomly on the Petri dish (Fig. 3A,C). We could thus determine whether this difference in terms of group behaviour affects the behavioural expression of learned behaviour of the target individuals. Given that training could be either such that AM was presented in a paired or in an unpaired way with the reward, this resulted in the four individual–group combinations displayed in Fig. 3B,D. The critical comparison is whether individual target larvae that had undergone the same training would behave differently dependent on *what* the group around it was doing: expressing innate odour preference or not.

### Experiment 4

**Fig. 4.**
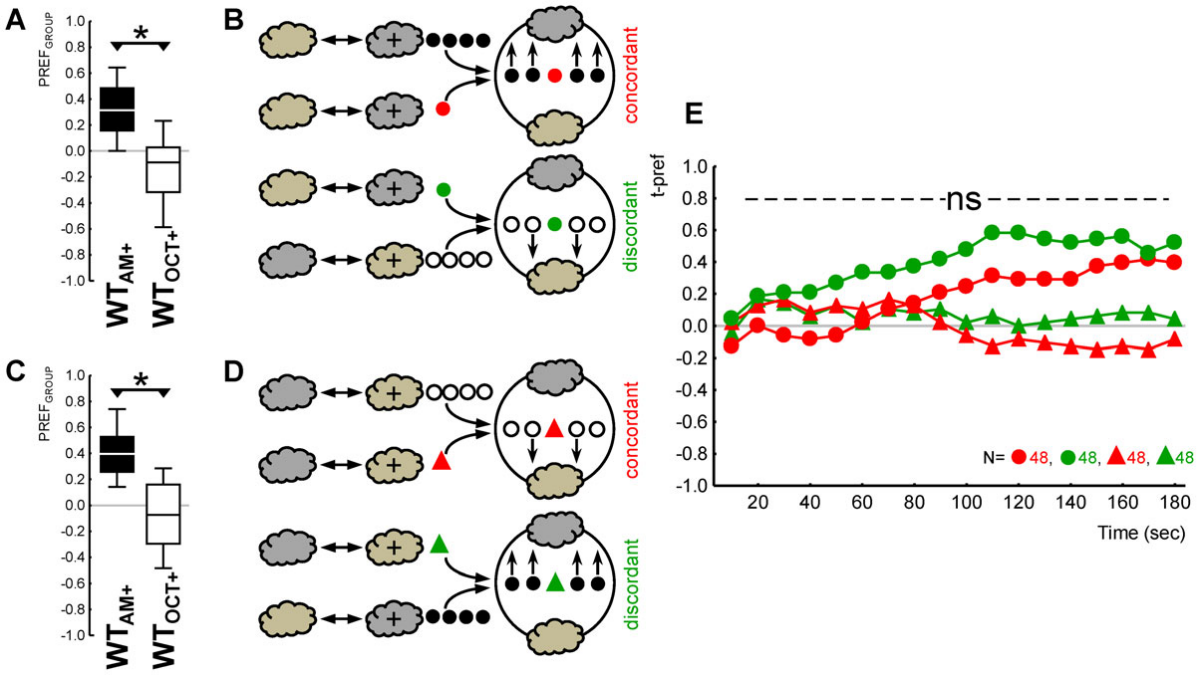
Learned olfactory behaviour of target individuals unaffected by learned behaviour of peer group. (A,C) Learned differences of group behaviour: groups of AM-rewarded larvae (filled plots) showed stronger AM-preference than OCT-rewarded groups (open plots). (B,D) Sketches of the experimental principle. (B) Target individuals (coloured circles) that had received AM-rewarded training were tested either embedded in a group of concordantly trained larvae (filled symbols) (this was the case for the target individuals symbolized by red circles) or in a group of discordantly trained larvae (open symbols) (green-circle target individuals). (D) Target individuals that had received OCT in a rewarded way were tested either in a concordantly or in a group of discordantly trained larvae (red and green triangles indicating the respective target individuals). The ns symbol refers to P>0.05 in MWU tests in both these respective ‘red versus green’ comparisons. The grey cloud indicates the odour AM, the brown cloud the odour OCT. Other details are as in the legend for Fig. 1.

Lastly, we tested whether the expression of learned olfactory behaviour by a target individual is affected by *what* likewise learned olfactory behaviour the group around it is expressing. Towards this end, the target individual was tested in groups that had either been trained concordantly to it, or that had been trained discordantly. For example, differently labelled pools of 30 WT larvae each underwent either OCT/AM+ training or AM/OCT+ training as in Experiment 2. For the concordant conditions (red symbols in Fig. 4), a single AM-rewarded target individual was placed into a likewise AM-rewarded group and olfactory preference was determined as described for Experiment 2. For the discordant conditions (green symbols in Fig. 4), AM-rewarded target individuals were tested in OCT-rewarded groups. In other words, the target individuals labelled red and green in the sketch of Fig. 4B had undergone the same training, yet were tested in either a concordantly-trained (red) or a discordantly trained (green) group; the same applies to Fig. 4D. Note that OCT-rewarded target individuals were likewise tested either con- or discordantly.

Given that, as intended, the AM-rewarded groups showed a stronger AM-preference than the OCT-rewarded groups (Fig. 4A,C), this allowed us to see whether this learned difference in odour preference of the groups would affect the behavioural expression of learned behaviour in the target individuals.

### Statistics

Nonparametric statistics were used throughout. For comparisons between experimental conditions, Mann–Whitney-U tests (MWU) were applied using Statistica, version 11 (StatSoft, Inc.). For comparisons of single experimental conditions to chance levels, one-sample sign tests as provided on http://www.fon.hum.uva.nl/Service/Statistics/Sign_Test.html were used. Comparisons were performed using PREF_GROUP_ or i-pref for analysing the behaviour of the groups or the target individuals, respectively.

Whenever multiple comparisons were made within one experiment, the significance level of P<0.05 was divided by the number of comparisons made; this maintained an experiment-wide error rate of 5% (Bonferroni-correction).

In box plots, the middle line indicates the median, the box boundaries the 25%/75% quartiles, and the whiskers the 10%/90% quantiles.

## RESULTS

### Experiment 1

We embedded experimentally naïve target individuals into groups of larvae of either WT or the anosmic *Orco^1^* genotype and offered them a choice between an AM-scented and a non-scented side of a Petri dish. We could thus test whether there were any behavioural differences between the target individuals embedded in a WT group that did show attraction to the odour (filled box plots in Fig. 1A,D: OSS tests, P<0.05/2, N = 84, 50) or an *Orco^1^* mutant group that did not (open box plots in Fig. 1A,D: OSS tests, P>0.05/2, N = 84, 50) (between-group comparisons: MWU tests, P<0.05, U = 1025.0 and U = 161.0, for Fig. 1A and Fig. 1D, respectively). We found no differences in the behaviour of WT target individuals embedded in a WT group versus in an *Orco^1^* mutant group (red versus green symbols in Fig. 1C; MWU test, P = 0.99, U = 3526.5). Likewise, anosmic *Orco^1^* mutant target larvae behaved indistinguishably in a WT group and in an *Orco^1^* mutant group (red versus green symbols in Fig. 1F; MWU test, P = 0.36, U = 1118.0).

We conclude that it does not matter to the behaviour of an experimentally naïve target individual whether a group of surrounding and likewise experimentally naïve larvae shows olfactory preference behaviour – or not. The general implication of this result is that individual behaviour is independent of group behaviour. Specifically, it implies that group-based measures of olfactory function neither are degraded by smell-blind animals distracting smell-competent ones, nor are inflated by smell-blind animals following their olfactory able peers.

### Experiment 2

Next, we approached the question of ‘peer pressure’ in larval *Drosophila* from a different angle: we tested olfactory choice behaviour between two odours in experimentally naïve target individuals embedded in groups of larvae that had undergone differential training with these two odours (Fig. 2B). That is, groups of WT larvae were either rewarded in conjunction with AM but not with OCT (OCT/AM+), or were trained reciprocally (OCT+/AM). We then embedded experimentally naïve WT target individuals in these reciprocally trained groups and recorded choice behaviour, both of the groups, and of the target individuals.

Differential training of the groups was successful: AM-rewarded groups showed higher AM-preference than OCT-rewarded groups (Fig. 2A: MWU test, P<0.05, U = 1059.5, N = 117, 119). This differential training effect, as reported previously under similar conditions (e.g. Saumweber et al., 2011; see also Scherer et al., 2003), manifested at a baseline level of a slight preference for AM and thus as a strong attraction to AM in the AM-rewarded group (Fig. 2A: OSS test, P<0.05/2) and a weak attraction to OCT in the OCT-rewarded group (Fig. 2A: OSS test, P>0.05/2). In any event, for the purpose of the current study it was only critical that the differentially trained groups did differ in their learned olfactory choice behaviour, such that we could ask whether experimentally naïve target individuals would be affected by these differences. This was not the case: olfactory choice of the target individuals was statistically indistinguishable when embedded in an AM-rewarded versus in an OCT-rewarded group (Fig. 2C; MWU test, P = 0.94, U = 6925.0).

We conclude that the learned olfactory behaviour of a group of larvae does not matter to the behaviour of an experimentally naïve target individual embedded in such a group. The general implication of this result again is that individual behaviour is independent of group behaviour. Specifically, it implies that group-based measures of associative memory are not inflated by non-learner individuals following their mnemonically able peers.

### Experiment 3

In the previous two experiments we found that innate odour preference of a target individual was not influenced by what a group of surrounding larvae was doing. In the next two experiments, we asked whether a group could exert such influence upon the learned odour preference of a target individual. This is not trivial, given that innate and learned olfactory behaviour draw on different 3^rd^ order neuronal pathways, and because learned, but not innate, olfactory behaviour is affected by the presence of reinforcement during the test (see Introduction for more details).

Differential group behaviour was implemented by using either WT or anosmic *Orco^1^* mutants in a choice situation with one side of a Petri dish equipped with an odour (AM) and the other with an empty odour container (EM). Under such conditions, the WT group approached the odour, whereas the *Orco^1^* mutant group did not (Fig. 3A: OSS tests, P< and >0.05/2 for either genotype, N = 40, 40; a between-group comparison with a MWU test yields P<0.05, U = 389.5) (Fig. 3C: OSS tests, P< and >0.05/2 for either genotype, N = 40, 40; MWU test P<0.05, U = 469.5). Did this difference of group behaviour affect the expression of learned behaviour in target individuals? This is not the case: AM-rewarded target individuals behaved indistinguishably when embedded in an odour-preferring WT group versus in an anosmic *Orco^1^* mutant group (Fig. 3E, circles: MWU test, P = 0.64, U = 751.5). The same held true for target individuals that had received AM in a non-rewarded way (Fig. 3E, triangles: MWU test, P = 0.80, U = 774.5).

We conclude that it does not matter for the expression of learned olfactory behaviour of a target individual whether a group of surrounding larvae expresses olfactory preference – or not. The general implication of this result, once more, is that individual behaviour is independent of group behaviour. Specifically, it implies that learner individuals are not distracted by the level of innate olfactory preference in their peers.

### Experiment 4

Lastly, we asked whether learned olfactory behaviour of a target individual was affected by the kind of learned behaviour a surrounding group of larvae was expressing. Differences in group behaviour were implemented by differential training: one set of groups received OCT/AM+ training (filled symbols in Fig. 4A–D) while the second set of groups was trained reciprocally (AM/OCT+; open symbols in Fig. 4A–D). This, as intended, introduced clear differences in olfactory choice behaviour between these sets of groups (Fig. 4A,C: MWU tests, P<0.05, U = 228.5 and 196.5, respectively; all N = 48).

Target individuals were trained with, for example, rewarded presentations of AM and unrewarded presentations of OCT (OCT/AM+) (coloured circles in Fig. 4B). These target individuals were then tested for their odour choice behaviour when embedded either in a group of larvae that had undergone the same training regimen, which in other words had been trained concordantly (red circles in Fig. 4B), or embedded in a discordantly trained group, that is in a group that had undergone AM/OCT+ training (green circles in Fig. 4B). It turned out that the target individuals behaved indistinguishably when embedded in a concordantly versus in a discordantly trained group (Fig. 4E, circles: MWU test, P = 0.13, U = 946.5). Please note a trend, however, for the discordantly tested target individuals having a *stronger* preference for the odour they had been rewarded with, namely in this case for AM (green circles in Fig. 4E).

In the case of the OCT-rewarded target individuals (triangles in Fig. 4D) there likewise was no significant difference in learned olfactory behaviour in the concordantly tested case (red triangles in Fig. 4D) versus in the discordantly tested case (green triangles in Fig. 4D) (MWU test, P = 0.70, U = 1100.5). Please note a trend for the discordantly tested target individuals having a *weaker* preference for the odour they had been rewarded with, namely for OCT in this case (green triangles in Fig. 4E).

We conclude that target individuals express learned olfactory behaviour regardless of whether a surrounding group of larvae expresses that same or a different type of learned behaviour. Once more, the general implication of this result is that individual behaviour is independent of group behaviour. Specifically, it implies that learner individuals are not affected by the expression of learned behaviour in their peers.

## DISCUSSION

We provide an analysis into whether the olfactory preference of individual target larvae is modulated by what a group of surrounding larvae is doing. For none of the four tested combinations of innate and learned olfactory behaviour this is the case:

- Innate olfactory preference of a target individual is neither affected by the level of innate olfactory preference in the surrounding group (Fig. 1) nor by the expression of learned olfactory preference in the group (Fig. 2).
- Likewise, learned olfactory preference of a target individual is neither affected by the level of innate olfactory preference of the surrounding group (Fig. 3), nor by the learned olfactory preference the group is expressing (Fig. 4).

We conclude that, to the extent tested, the olfactory behaviour of individuals, be it innate or learned, is independent of what surrounding larvae are doing, and that in this sense there is no social interaction in larval *Drosophila*.

It remains to be seen how robust this conclusion will be with respect to parametric variations in the experiment, such as larval density or the ratio of ‘informed/non-informed’ individuals, which is 1/29 throughout the current study. We did not attempt to systematically explore this parameter space; rather, our choice of parameters was specifically tailored to the conditions of a widely used learning paradigm (described, amongst others, in Scherer et al., 2003; Selcho et al., 2009; Apostolopoulou et al., 2013). The absence of social interaction effects as reported here thus eases worries that learning scores in this paradigm were inflated by good learners or were underestimated by poor learners affecting their peers. As a corollary, one likewise does not need to reckon with the possibility of mutant phenotypes in learning actually reflecting deficiencies in social interaction.

Regarding innate olfactory behaviour in larval *Drosophila*, our findings from Experiment 1 are in line with the results obtained by Kaiser and Cobb (Kaiser and Cobb, 2008). The authors exposed wild-type larvae to pentyl acetate (thus rendering them unresponsive to pentyl acetate in a preference test: “adapted”), or not (thus keeping them responsive to pentyl acetate: “non-adapted”). Comparing homogeneous groups of adapted or non-adapted larvae to mixed groups of adapted/non-adapted larvae, the performance scores of the adapted or non-adapted subgroups in the mixed population were indistinguishable from the respective homogeneous testing conditions. These findings indicate that each individual makes its own odour choice, uninfluenced by *what* their peers are doing. In a different approach, the authors looked into whether the mere presence (versus absence) of a larval group affects the odour preference of the target individual. Half of the individuals underwent the test alone; half of the individuals were tested in the presence of a group of other larvae. The odour preferences of individuals tested alone versus in a group were found to be indistinguishable, indicating that the presence of other larvae has no bearing on preference scores.

Regarding learned behaviour in larval *Drosophila*, Kohn et al. asked whether the mere presence of a group of other larvae has an effect on a target individual (Kohn et al., 2013). They report that larvae carrying the sitter allele of the *foraging* gene (CG: CG10033), but not the ones carrying the rover allele, showed higher memory performance in group assays as compared to individual assays. This is consistent with our results if one supposes that either in our fly cultures the rovers were outnumbering the sitters, or if the facilitating effect of the group in the sitters that Kohn et al. had observed (Kohn et al., 2013) was related to the mere presence of the group, rather than to what that group was doing.

Regarding learned olfactory behaviour in adult *Drosophila*, Quinn et al. in a classical experiment trained two populations of adult flies with electric shock to avoid different odours (Quinn et al., 1974). The authors then either (i) tested those populations separately, or (ii) mixed those populations and tested whether they separate according to their respective training experiences. It was found that the populations do indeed separate according to previous training experience, but memory scores were somewhat lower compared to the tests where all flies had experienced identical training. The results argue that choice chiefly resides in the individual fly; the degree to which choice is modulated by *what* surrounding flies are doing remained unclear, however.

Using a presence–absence comparison, Tempel et al. looked into how the presence of other flies affects the testing performance of individuals (Tempel et al., 1983). Using odour-sugar learning, the authors trained small groups of 5–8 flies simultaneously but tested them individually, one by one. It turned out that learning scores were about as high as those observed in the normal mass-group learning procedure that uses groups of around 40 flies in training and testing. Again, this supports the case that flies choose the odour independently of each other, as the presence of other flies during testing does not alter memory performance. This experiment did not ask whether, beyond the mere presence, flies take into account *what* the other flies do.

A recent paper by Chabaud et al., also dealing with learned olfactory behaviour in adult flies, includes experiments that utilize both the comparison of individual testing versus mass testing and of testing target individuals in differentially behaving groups (Chabaud et al., 2009). The authors report that after training in a group, flies showed stronger memory when also tested in groups than when tested individually. The presence of a trained group during test therefore appeared to improve individuals' memory recall. Interestingly, this facilitating effect was exerted only by trained but not by untrained, naïve groups. Thus, trained individuals receive cues from trained groups to improve their performance, but not from untrained groups.

In an altogether different, ecologically inspired approach Durisko and Dukas allowed their larvae to choose between food patches of different quality placed on opposite sides of a Petri dish (Durisko and Dukas, 2013). On one side the food was ‘fresh’ while on the other side it was ‘used’, that is it had previously been occupied by other larvae for several hours. Under these conditions, the larvae significantly preferred the side with the used food. This preference was not increased if the used food patch, rather than being cleared of larvae before the test as in the previous experiment, was still occupied by larvae – arguing it is the past-presence of the larvae, rather than their actual presence, that matters most for preference behaviour (please note that these experiments do not ask whether the kind of behaviour of other larvae is recognized by an individual). Correspondingly, if larvae were offered a choice between a fresh food patch unoccupied by larvae and a fresh food patch added with larvae at the time of testing, no difference in preference was detected. Thus, larvae show innate attraction to cues from a used food patch, but not to the acute presence of larvae occupying it. In a follow-up associative learning experiment, the authors paired an odour A with unoccupied fresh food, while the alternative odour B was paired with either (i) unoccupied used food, (ii) occupied used food, or (iii) occupied fresh food. Larvae preferred the alternative odour B over A after all three kinds of training (note that for the experimental condition [iii] this implies that the occupancy of the fresh food does influence its reward function, although as argued before it did not affect preference behaviour; similar dissociations have been reported previously regarding salt, sugar, and quinine [Niewalda et al., 2008; Schipanski et al., 2008; El-Keredy et al., 2012; Apostolopoulou et al., 2014]). That unoccupied fresh food is apparently the least powerful reward appears plausible, because in a Petri dish assay digging into the substrate, as part of the larvae's attempts to find shelter, is easier if the food is softened by the past-presence or is cracked-up by the efforts of acutely present other larvae (or of any other agent of decay, we hesitate to add). In any event, these analyses concern effects of the presence versus absence of other larvae, and do not speak to the question whether larvae recognize what other larvae are doing.

In summary, it seems clear from the literature that the past as well as acute presence of conspecifics does matter to the behaviour of larval as well as of adult *Drosophila*. It remains to be uncovered whether and to what extent these effects are mediated by species-specific signals, cues from the larval microbiome (Venu et al., 2014), or by more generic cues. However, neither our present experiments, nor the literature, offer conclusive evidence that in the context of various types of olfactory learning or choice experiments individual *Drosophila* would take note of *what* surrounding conspecifics are doing. Such type of social interaction rather may be limited to situations that are defined as social to begin with, such as aggression (Vijendravarma et al., 2013) and, in adults at least, courtship (Villella and Hall, 2008). Indeed, it is tempting to speculate that the requirement for social coordination in courtship may be the base on which to evolve other forms of interaction among conspecifics.
